# Habitat fragmentation enhances microbial collective defence

**DOI:** 10.1098/rsif.2024.0611

**Published:** 2025-02-12

**Authors:** Nia Verdon, Ofelia Popescu, Simon Titmuss, Rosalind J. Allen

**Affiliations:** ^1^ Theoretical Microbial Ecology, Institute of Microbiology, Faculty of Biological Sciences, Friedrich Schiller University, Jena, Germany; ^2^ Cluster of Excellence Balance of the Microverse, Friedrich Schiller University, Jena, Germany; ^3^ School of Physics and Astronomy, University of Edinburgh, James Clerk Maxwell Building, Peter Guthrie Tait Road, Edinburgh EH9 3FD, UK

**Keywords:** stochastic population dynamics, antibiotic resistance, microbial ecology and evolution, habitat fragmentation

## Abstract

Microbes often inhabit complex, spatially partitioned environments such as host tissue or soil, but the effects of habitat fragmentation on microbial ecology and infection dynamics are poorly understood. Here, we investigate how habitat fragmentation impacts a prevalent microbial collective defence mechanism: enzymatic degradation of an environmental toxin. Using a theoretical model, we predict that habitat fragmentation can strongly enhance the collective benefits of enzymatic toxin degradation. For the example of β-lactamase-producing bacteria that mount a collective defence by degrading a β-lactam antibiotic, we find that realistic levels of habitat fragmentation can allow a population to survive antibiotic doses that greatly exceed those required to kill a non-fragmented population. This ‘habitat-fragmentation rescue’ is a stochastic effect that originates from variation in bacterial density among different subpopulations and demographic noise. We also study the contrasting case of collective enzymatic foraging, where enzyme activity releases nutrients from the environment; here we find that increasing habitat fragmentation decreases the lag time for population growth but does not change the ecological outcome. Taken together, this work predicts that stochastic effects arising from habitat fragmentation can greatly enhance the effectiveness of microbial collective defence via enzymatic toxin degradation.

## 1. Introduction

**Figure 1.**
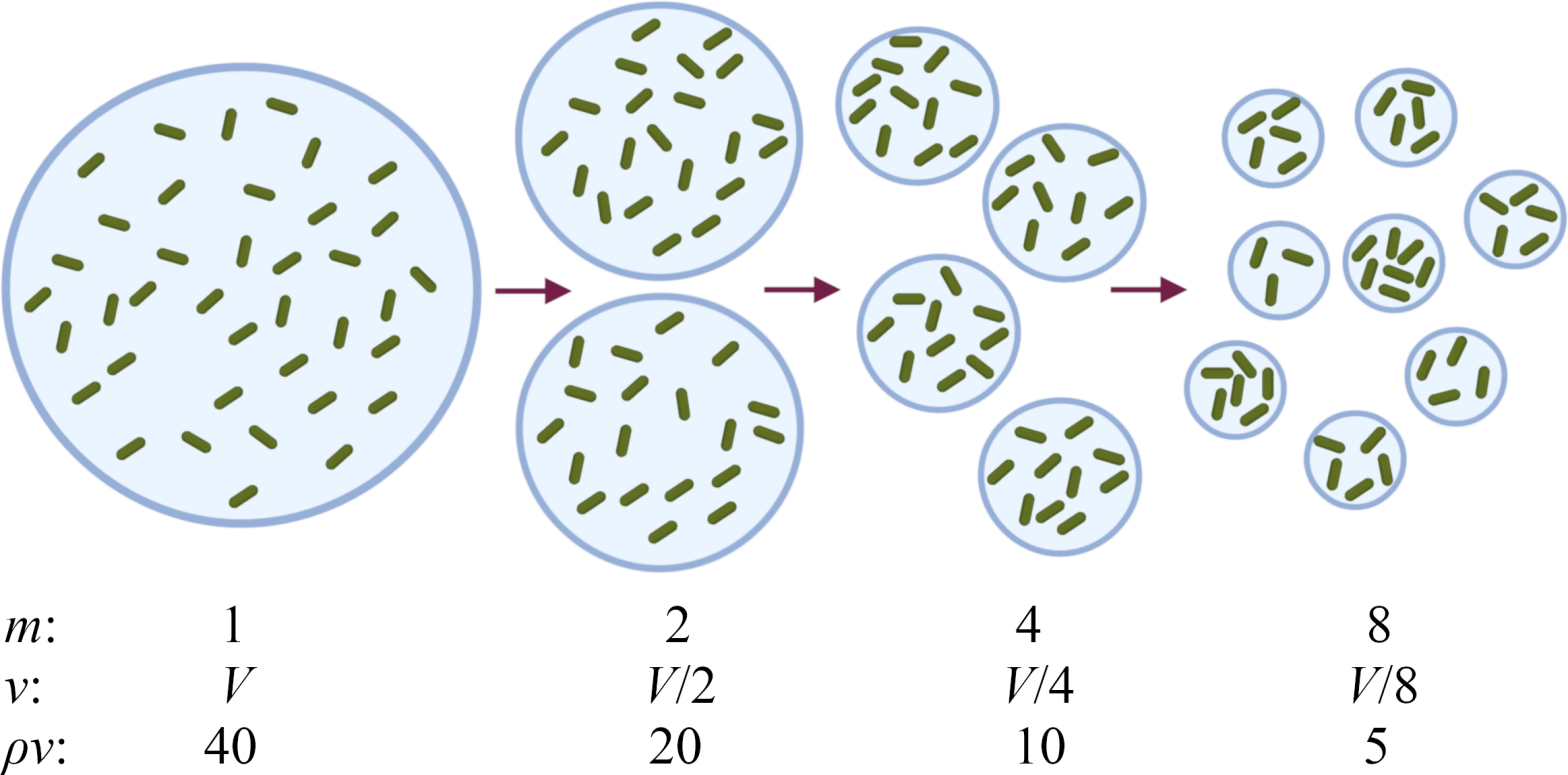
Modelling habitat fragmentation by systematically partitioning a microbial population. A population of microbes of density ρ, contained in volume V, is partitioned into smaller sub-volumes of volume v=V/m. For degree of partitioning m, the average number of microbes per sub-volume is ρv. Microbes are distributed stochastically among the sub-volumes, so that the number of microbes in a given sub-volume follows a Poisson distribution. As an exemplar, in this diagram, we use ρv= 40 for m = 1.

Microbial communities commonly inhabit spatially fragmented habitats, such as particles or aggregates in aqueous environments [1,2], plant roots [3] or leaf surfaces [4,5], crypts in the gut lining [6], pores in the soil [7,8], in the skin [9] or, for intracellular pathogens, the interior of host cells [10]. Even in the absence of physical habitat fragmentation, slow diffusion of nutrients and/or signals can lead to effective fragmentation [11]. Habitat fragmentation has been found to significantly affect microbial interactions [12–15], community assembly [16,17] and population dynamics [18,19], but mechanistic understanding of these effects is lacking. The absence of mechanistic models for microbial interactions in fragmented habitats means that predictions are often made by extrapolation from well-mixed habitats. Here, we investigate the mechanisms by which habitat fragmentation can alter microbial interactions, by developing a mathematical model in which a microbial population is systematically partitioned into smaller sub-habitats, while keeping the total population size and volume the same (figure 1). Using this approach, we show how habitat fragmentation can strongly affect a prevalent microbial ecological trait: collective defence by enzymatic degradation of an environmental toxin.

Many microbes produce enzymes that modify their local environment; examples include antibiotic-degrading enzymes such as β-lactamases [20], cellulases that allow digestion of plant matter [21], hydrolases that degrade marine polysaccharides [22], sucrose digestion by yeast invertases [23] and the production of virulence factors such as elastase that cause tissue damage [24]. Enzymatic modification of the environment is a collective behaviour, since the effects are shared by nearby microbes. Indeed, enzyme production has been analysed in the framework of social evolution theory, which addresses how enzyme production can be maintained evolutionarily in the face of cheater non-producers who reap the benefits but do not pay the cost of enzyme production [23,25–29]. For mixed populations of cooperators and cheaters, habitat fragmentation can favour the evolution of cooperators through kin selection, although it may also increase the level of local competition between cooperators [30].

Here, we consider a clonal population, composed entirely of enzyme producers (i.e. cooperators). We focus on the well-studied example in which antibiotic-resistant bacteria produce β-lactamase enzymes that degrade β-lactam antibiotics [20,31]. Consistent with the concept of β-lactamase production as a collective defence strategy against the antibiotic, β-lactamase-producing bacteria often show an inoculum effect, whereby populations with high initial bacterial density survive, while those with low initial density are killed [28,32,33]. Recent mathematical modelling has shown that this effect can be understood as a race for survival, which depends on the relative timescales for antibiotic killing versus antibiotic degradation [34]. However, the role of habitat fragmentation has not yet been considered.

We present a theoretical modelling approach, which we use to predict the effects of habitat fragmentation on microbial enzyme producers. Our main result is that, for microbes that engage in collective defence through enzymatic degradation of a toxin (such as β-lactam degradation), habitat fragmentation can dramatically increase the probability of survival. This effect is intrinsically stochastic, arising from the variability in initial population densities among the partitioned subpopulations, as well as from demographic stochasticity in microbial growth and killing dynamics. We also study a contrasting model in which a microbial population uses enzymes to boost growth by releasing nutrients from the environment. For this case, we find that the effects of fragmentation are much weaker.

Enzymatic modification of the environment by microbes is ubiquitous, from clinical infections to biotechnology and biogeochemistry [20–24,35,36]. Our study shows how the intrinsic stochasticity associated with habitat fragmentation can alter the ecology of these interactions.

## 2. Results

### 2.1. Collective defence through enzymatic degradation

We consider a microbial population that is exposed to an environmental toxin—as a specific example, a population of β-lactamase-producing bacteria exposed to a β-lactam antibiotic. We propose a conceptually simple model in which the population (contained in volume V) grows exponentially if the antibiotic concentration is below a threshold $a_{th}$ but dies exponentially if the antibiotic concentration is above the threshold. The threshold concentration $a_{th}$ corresponds to the single-cell minimum inhibitory concentration (scMIC)^1^ [37]. In addition, the microbes produce antibiotic-degrading β-lactamase enzyme: this causes the antibiotic concentration to decrease with time. The microbial population size N(t) is described by the following dynamical equation:


(2.1) $$\begin{array}{lcr} & \dot{N(t)}=N(t)\left[\mu \theta (a_{th}-a)-\gamma \theta (a-a_{th})\right]\;, & \end{array}$$

where θ(x) denotes the Heaviside step function (θ(x)=1 if x≥0 and zero otherwise), μ is the growth rate for low antibiotic $a(t)<a_{th}$, and γ is the death rate for high antibiotic $a(t)>a_{th}$. The antibiotic is degraded according to


(2.2) $$\begin{array}{lcr} & \dot{a(t)}=-\frac{bN(t)}{V}\left(\frac{r_{max}a(t)}{a(t)+K_{M}}\right)\;, & \end{array}$$

where b is the number of enzyme molecules per cell (i.e. the enzyme concentration is bN/V) and each enzyme degrades antibiotic according to Michaelis–Menten kinetics with parameters $r_{max}$ and $K_{M}$ (note that if the enzyme remains partially or wholly within the bacterial cell, the $r_{max}$ parameter also implicitly accounts for antibiotic transport across the cell boundary [34,38]). We assume that the initial antibiotic concentration, $a_{\mathrm{init}}$, is high, $a_{\mathrm{init}}>a_{th}$. Our model is designed to be simple and neglects many factors, including the details of antibiotic growth inhibition and killing dynamics [39], and depletion of antibiotic by irreversible binding to its target [31]. Conceptually similar but more detailed models have been proposed in previous work [26,29,34] and lead to qualitatively equivalent results [34].

**Figure 2.**
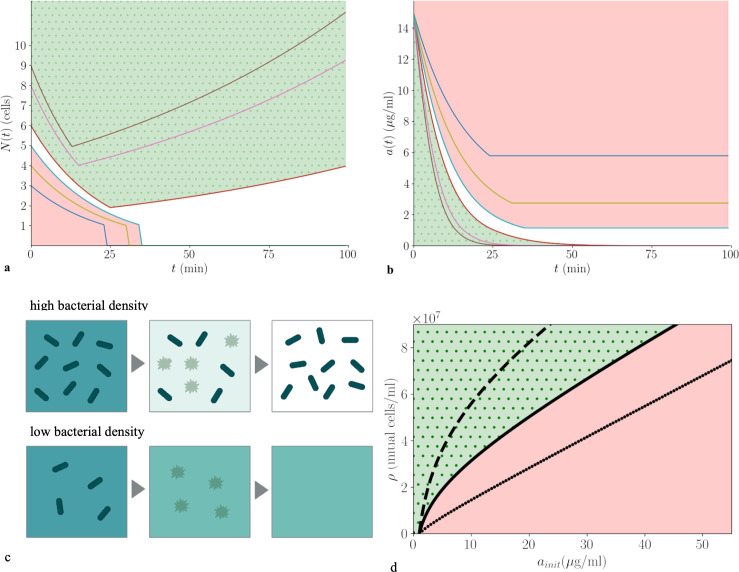
Collective enzymatic defence: population density threshold for survival. (*a,b*) Predictions of the model, equations (2.1) and (2.2), for the microbial population size N(t) and the antibiotic concentration a(t) ((*a*) and (*b*), respectively), for a range of values of the initial microbial population size $N_{\mathrm{init}}$ (coloured lines; $N_{\mathrm{init}}$ ranges between 3 and 9). The colour coding is consistent between (*a*) and (*b*). The red-shaded region indicates death of the microbial population; the green-shaded region indicates the survival and regrowth of the population. The parameters are as follows: $br_{max}=3.5\times 10^{-8}$μg cell^−1^ min^−1^ [29], $K_{M}=6.7$μg ml^−1^ [29], $a_{th}=1$ μg ml^−1^ [29], $a_{\mathrm{init}}=15$ μg ml^−1^, μ=0.01 min^−1^ [40], γ=0.045 /min^−1^ [29], V=100 pl [40]. (*c*) Schematic showing the two ecological outcomes. The background colour indicates antibiotic concentration (dark = high, light = low) while the dark green objects represent bacteria, which can be alive (rod-shaped) or dead (faint stars). At high bacterial density, antibiotic is degraded below the threshold $a_{th}=1$ before the population is entirely killed; therefore, the population recovers; at low bacterial density, the entire population is killed before the antibiotic concentration reaches the threshold. (*d*) Phase diagram showing the predicted outcome (killing or survival and regrowth; red and green, respectively) as a function of initial bacterial density ρ and initial antibiotic concentration $a_{\mathrm{init}}$. The lines indicate the critical density $\rho^{*}$, equation (2.3), for different values of the Michaelis–Menten parameter $K_{M}$: solid line $K_{M}=6.7$ μg ml^−1^ [29], dashed line $K_{M}=15$ μg ml^−1^, dotted line $K_{M}=1$ μg ml^−1^.


In our model, two distinct outcomes are possible (figure 2*a*,*b*). The microbial population may be killed outright, or it may reduce the antibiotic concentration below the threshold $a_{th}$ and then regrow. This phenomenon has previously been described as a race for survival, since the outcome depends on the relative timescales of killing and antibiotic degradation—figure 2*c* [34]. The population will survive and regrow if its initial density is greater than a critical value $\rho^{*}$ that depends on the initial antibiotic concentration [34],

(2.3) $$\begin{array}{lcr} & \rho^{*}=\frac{\gamma }{br_{max}}\left[a_{\mathrm{init}}-a_{\mathrm{th}}+K_{M}\ln\;\left(\frac{a_{\mathrm{init}}}{a_{\mathrm{th}}}\right)\right]\;. & \end{array}$$

(For derivation of equation (2.3), see electronic supplementary material, Supplementary Theoretical Derivations, Eqs. S6–S14.) The line $\rho^{*}(a_{\mathrm{init}})$ defined by equation (2.3) defines an ecological phase boundary that separates regions of parameter space where the microbial population is killed from regions where it survives and regrows (figure 2*d*).

The shape of the ecological phase boundary reveals an inoculum effect: populations with a higher initial density can survive at higher initial antibiotic concentrations [32] (figure 2*d*). This arises from the collective nature of antibiotic degradation [26,28,29,34]. The qualitative nature of the inoculum effect depends on the kinetic parameters of the enzyme. If $K_{M}$ is small ($a_{init}\gg K_{M}$), the phase boundary is linear (figure 2*d*, dots), but if $K_{M}$ is large ($a_{init}\ll K_{M}$), it becomes logarithmic (figure 2*d*, dashes; equation (2.3)).

### 2.2. In a fragmented habitat, subpopulation killing is stochastic

**Figure 3.**
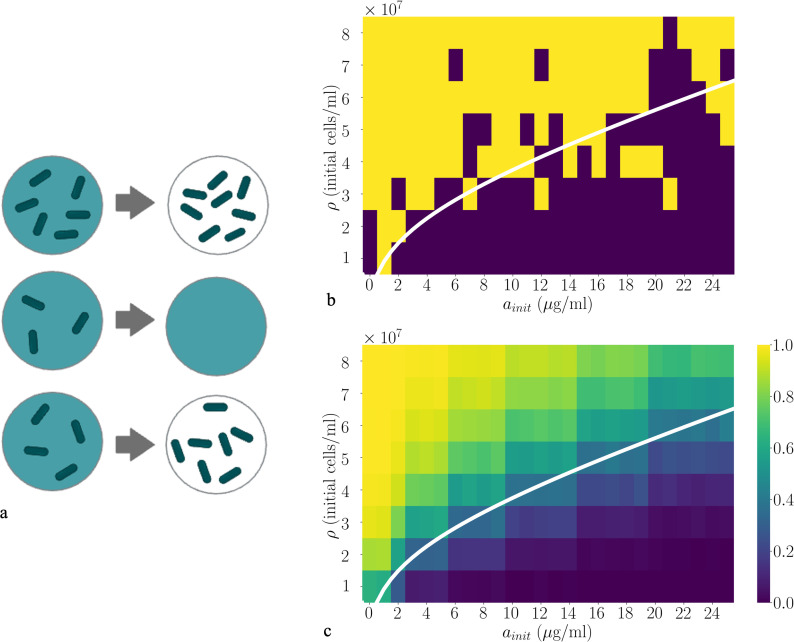
Subpopulation killing is stochastic. (*a*) Illustration that different replicate subpopulations experience different ecological outcomes depending on their stochastic initial population density. The background colour represents antibiotic concentration (dark = high, light = low) while the dark green objects represent live bacteria. A subpopulation that is below the threshold density for survival $\rho^{*}$ is killed, while subpopulations with higher initial density survive and grow. (*b*) The model of equations (2.1) and ((2.2) is simulated for a single subpopulation with Poisson distributed initial population size, for a range of values of the initial antibiotic concentration $a_{init}$ and the mean population density ρ, with v=100 pl. The other parameters are $br_{max}=3.5\times 10^{-8}$ μg cell^−1^ min^−1^, $K_{M}=6.7$ µg ml^−1^, $a_{th}=1$ µg ml^−1^, μ=0.01 min^−1^, γ=0.045 min^−1^ (as in figure 2). Simulation runs where the subpopulation is killed are shown as dark squares; those where the subpopulation survives and regrows are shown as light squares. (*c*) Equivalent simulations to those of (*b*), averaged over 1000 subpopulations for each condition. Here, the probability $p_{s}$ of subpopulation survival is indicated by the colour scale. In both (*b*) and (*c*), the ecological phase boundary corresponding to $\rho^{*}(a_{\mathrm{init}})$ from figure 2*d* is shown as a white line. For a detailed description of the simulation methods, see electronic supplementary material, Supplementary Numerical Simulation Methods.

We now ask how habitat fragmentation (e.g. within skin pores or crypts in the gut lining) affects the fate of a microbial population engaged in collective defence, under lethal conditions where a well-mixed population would be killed. Using as our example a population of β-lactamase-producing bacteria exposed to an antibiotic concentration well above the MIC, we now suppose that the total volume V, containing a population of density ρ, is partitioned into m sub-volumes of volume v=V/m [19] (figure 1). The average (initial) number of microbes per sub-volume is then ${\bar{N}}_{\mathrm{init}}=\rho v$. However, the sub-volumes are filled stochastically. Here, we assume that the number of microbes $N_{i,\mathrm{init}}$ in sub-volume i is sampled from a Poisson distribution with probability $p(N_{i,\mathrm{init}})=e^{-\rho v}(\rho v{)}^{N_{i,\mathrm{init}}}/N_{i,\mathrm{init}}!$. Poisson statistics have indeed been observed for encapsulation of bacteria in microfluidic droplets [40–42]; however, other statistical distributions for the number of microbes per sub-volume could arise, for example, from spatial habitat fragmentation. As we discuss later, we would expect similar results in this case. We also assume that each sub-volume also contains antibiotic at uniform concentration $a_{\mathrm{init}}$. Neither microbes nor antibiotic can be exchanged between sub-volumes, so that the microbe–antibiotic dynamics evolve independently in each sub-volume. Because different sub-volumes have stochastically different initial subpopulation sizes, we anticipate different outcomes among replicate sub-volumes (figure 3*a*). We model the dynamics in each sub-volume deterministically through equations (2.1) and (2.2) (although later we also consider stochastic birth–death dynamics).

The relevant ecological outcome here is the survival versus killing of the fragmented microbial population—for example, in antibiotic treatment, even a few surviving microbes can regrow and cause disease recurrence. We therefore calculate the probability that the entire microbial population is killed, or its complement, the ‘survival probability’, which is the probability that any microbes within the population survive the treatment.

We first consider the subpopulation of microbes contained within a single sub-volume v. In our model, the fate of the subpopulation is determined by its initial density (equation (2.3) and figure 2*d*); it survives if $N_{i,\mathrm{init}}/v>\rho^{*}$. The probability $p_{s}$ for this condition is controlled by the Poisson distribution of initial subpopulation sizes: $p_{s}=e^{-\rho v}\sum_{j=N^{*}}^{\infty }(\rho v{)}^{j}/j!$, where $N^{*}=\left\lceil \rho^{*}v\right\rceil$. Indeed, numerical simulations of the model dynamics for a single subpopulation with Poisson distributed initial population size show stochastic outcomes (figure 3*a*,*b*). For antibiotic concentrations and (average) microbial densities below the ecological phase boundary, for which the bulk, non-fragmented population is killed (figure 2*d*), some of the stochastically simulated subpopulations survive. Conversely, above the phase boundary, where a bulk population survives, some stochastically simulated subpopulations are killed (figure 3*b*). Averaging these results over many stochastic simulation runs (figure 3*c*) highlights that there is a non-zero probability of survival in regions of parameter space that are below the phase boundary and, conversely, a non-zero probability of killing in regions above the phase boundary. Therefore, stochasticity in the initial population density can change the ecological outcome for individual subpopulations.

### 2.3. Habitat fragmentation can rescue a microbial population in a lethal environment

We now consider the fate of the entire microbial population, consisting of m independent subpopulations. The probability that the entire population is killed is given by


(2.4) $$\begin{array}{lcr} & 1-P_{s}=(1-p_{s}{)}^{m}\;, & \end{array}$$

where $P_{s}$ is the total population survival probability, i.e. the probability that any of the subpopulations survives and regrows.

**Figure 4.**
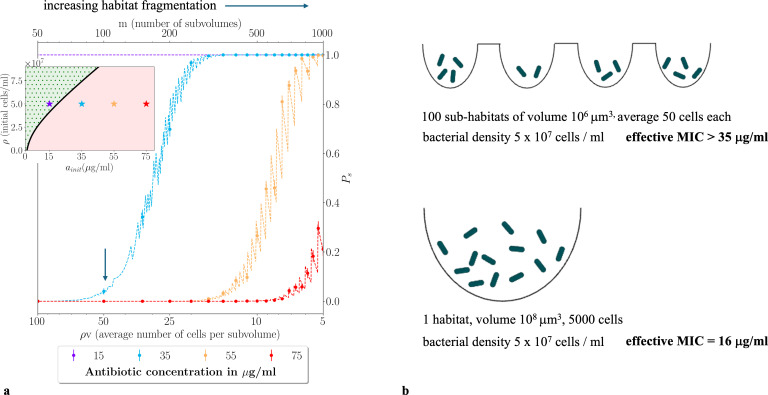
Habitat fragmentation can rescue a microbial population in a lethal environment. (*a*) Total population survival probability $P_{s}$ increases from zero to one as the habitat becomes more fragmented, for lethal parameter combinations for which a bulk population would be killed. $P_{s}$ is plotted as a function of m (top axis), or the average subpopulation size ρv (bottom axis), for a range of initial antibiotic concentrations $a_{init}$ (shown by colour) and mean population density $\rho =5\times 10^{7}$ cells ml^−1^. The data symbols show results of numerical simulations of the model of equations (2.1) and (2.2) with a Poisson distribution of initial subpopulation sizes (each data point is the mean of 1000 simulation runs; error bars show 95% confidence interval, assuming a binomial distribution), while the dashed lines show theoretical predictions obtained from equation (2.4). For the lowest antibiotic concentration (15 μg ml^−1^) the bacterial population always survives. For higher concentrations (35, 55 and 75 μg ml^−1^), the population is killed for low habitat fragmentation but starts to survive as the degree of habitat fragmentation increases. The arrow indicates the condition analysed in panel (*b*). The zig-zagging in the theoretical lines arises because of the discreteness of individual microbes, reflected in the bound $N^{*}=\left\lceil \rho^{*}v\right\rceil$ in the sum that features in the expression for $p_{s}$. The other parameters are $br_{max}=3.5\times 10^{-8}$μg cell^−1^min^−1 ^, $K_{M}=6.7$μg ml^−1^, $a_{th}=1$ μg ml^−1^, μ=0.01 min^−1^ and γ=0.045 min^−1^ (as in figure 2). The inset is included to show the equivalent outcomes for unfragmented populations exposed to the same antibiotic concentrations. The inset shows the phase diagram of figure 2, with the points (colour coded by antibiotic concentration) showing the location in the $\left(a_{init},\rho \right)$ plane relative to the survival phase boundary; a bulk, unfragmented population survives for 15 μg ml^−1^ but dies for 35, 55 or 75 μg ml^−1^ ($\rho^{*}$ values are $4.1\times 10^{7}$, $7.4\times 10^{7}$, $1.04\times 10^{8}$ and $1.3\times 10^{8}$cells ml^−1^, respectively). For a detailed description of the simulation approach, see electronic supplementary material, Supplementary Numerical Simulation Methods. (*b*) Consequences for treatment of fragmented versus non-fragmented infections. Using the same parameters as in (*a*), we compare a bacterial population of density $5\times 10^{7}$ cells ml^−1^ that is either fragmented into approximately 100 sub-habitats each of volume $10^{6}$ µm^3^ (top; indicated by the arrow in (*a*) or contained in an unfragmented habitat of volume approximately $10^{8}$ µm^3^ (bottom). The unfragmented population is predicted to have an effective MIC given by the phase boundary line in figure 2*d* (i.e. approximately 16 μg ml^−1^), while the fragmented population has a much higher effective MIC of 35 μg ml^−1^.

Habitat fragmentation strongly increases the probability of population survival. For conditions below the ecological phase boundary, for which a bulk population would be killed, simply fragmenting the population into sub-volumes (i.e. increasing m or equivalently decreasing ρv without changing the total population size or the total volume) can ensure its survival (figure 4*a*). Thus, for our β-lactamase example, the model predicts that a fragmented infection can survive antibiotic treatment doses that would be sufficient to kill a non-fragmented infection. To explore this point more explicitly, we model a β-lactamase-producing bacterial infection consisting of 5000 cells at density $5\times 10^{7}$ cells ml^−1^ in a volume $10^{8}$ μm^3^ (figure 4*b*). In an unfragmented habitat, the infection will be killed by antibiotic concentrations above the threshold given by the phase bounday line in figure 2*d*—for this cell density, this is approximately 16 μg ml^−1^ . However if the habitat is fragmented into 100 sub-habitats of volume $10^{6}$ μm^3^ (consistent with lung alveolae [43] or hair follicles ([44]), with on average 50 cells per sub-habitat, the model predicts that even an antibiotic concentration of 35 μg ml^−1^ is insufficient to eliminate the infection (figure 4*a*, arrow and figure 4*b*).

In our model, this ‘fragmentation rescue’ phenomenon arises from the stochastic distribution of microbes among the sub-volumes. Because the microbes are distributed stochastically, some sub-volumes have an initial population density higher than the survival threshold $\rho^{*}$ (i.e. above the phase boundary) even though the average density ρ is below the phase boundary. For a Poisson distribution of cell numbers, the coefficient of variation of the population density increases with the degree *m* of partitioning^2^; hence, the chance of survival is increased by habitat fragmentation. The same phenomenon would occur for any distribution of cell numbers where the CV increases with the degree of habitat fragmentation.

Three distinct antibiotic-killing regimes can be distinguished, for different degrees of habitat fragmentation. For low fragmentation (small m or large ρv in figure 4*a*), stochasticity is not important and the infection is killed ($P_{s}\approx 0$). As the degree of fragmentation increases, stochasticity becomes relevant and there is a significant probability of survival; here the model predicts an approximately linear relation between $\log\;P_{s}$ and the sub-volume size v (electronic supplementary material, Supplementary Text and figure S1). In the extreme fragmentation regime (very large m or very small ρv), the model predicts that the entire population survives, even for very high antibiotic concentration (figure 4*a*; see also electronic supplementary material, figure S3a). In this regime, the sub-volume size becomes small enough that the cell density exceeds the critical value $\rho^{*}$ even for a single microbe ($1/v>\rho^{*}$). However, our model is unlikely to be realistic in this limit (see electronic supplementary material, Supplementary text and figure S3 for further discussion).

For bacterial populations that do survive and regrow, spatial partitioning also influences the eventual population size, since subpopulations with stochastically higher initial population size start to regrow earlier (electronic supplementary material, Supplementary text and figure S2a).

### 2.4. Other sources of stochasticity can also lead to habitat-fragmentation rescue

So far, the only source of stochasticity in our analysis has been the random partitioning of microbes between subpopulations, since we used a deterministic model, equations (2.1) and (2.2), for microbial growth and death. However, other sources of stochasticity may be significant in small populations, including demographic stochasticity arising from the randomness of microbial birth and death events [45,46], and phenotypic or genotypic heterogeneity between individual microbial cells [47].

We explored the role of demographic stochasticity by including stochastic birth and death processes in our model (using a modified version of the Gillespie algorithm [48]; see the electronic supplementary material). Our simulations show that intrinsic stochasticity in the microbial killing dynamics can also rescue a population in a fragmented habitat (electronic supplementary material, figure S2) [49]. For the parameter set chosen here (representative of collective β-lactamase degradation in a microfluidic droplet set-up [40], the contribution of demographic stochasticity is approximately equal in magnitude to that of stochastic partitioning, with maximal effect when both sources of stochasticity are present (electronic supplementary material, figures S2 and S4).

### 2.5. Habitat fragmentation inhibits growth in a non-lethal environment

**Figure 5.**
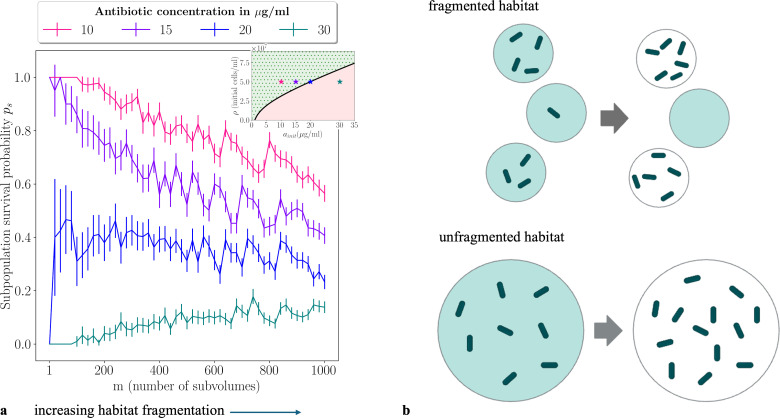
Habitat fragmentation is detrimental for microbes in a non-lethal environment. (*a*) The probability $p_{s}$ of survival for any given subpopulation, conditional on the sub-volume being initially occupied, is plotted as a function of the degree of habitat fragmentation m. The data are obtained from numerical simulations of the model of equations (2.1) and (2.2) with a Poisson distribution of initial subpopulation sizes. The mean population density $\rho =5\times 10^{7}$ cells ml^−1^ and the other parameters are $br_{max}=3.5\times 10^{-8}$ μg cell^−1^ ml^−1^, $K_{M}=6.7$ μg ml^−1^, $a_{th}=1$ μg ml^−1^, μ=0.01 min^−1^ and γ=0.045 min^−1^ (as in figure 2). The subpopulation survival probability decreases with habitat fragmentation for low initial antibiotic concentrations, i.e. for non-lethal conditions under which an unfragmented population would survive. The inset shows the equivalent outcomes for unfragmented populations exposed to the same antibiotic concentrations. The phase diagram of figure 2 is shown with the points (colour coded by antibiotic concentration) indicating the location in the $\left(a_{init},\rho \right)$ plane relative to the survival phase boundary; a bulk, unfragmented population survives for 10 and 15 μg ml^−1^, but 20 μg ml^−1^ is the threshold for survival of the bulk population, and it dies at 30 μg ml^−1^. (*b*) Illustration of how stochastic partitioning of microbes between sub-habitats decreases overall growth under (on average) non-lethal conditions. Subpopulations with low initial density are killed and do not contribute to population growth, even though the conditions are such that an unfragmented population survives.

Interestingly, the effects of habitat fragmentation are qualitatively different when the environment is non-lethal, i.e. for low antibiotic concentrations or high cell densities, above the phase boundary for survival, $\rho >\rho^{*}$ (green region of figure 2*d*). In this case, habitat fragmentation is actually detrimental to the bacterial population. Our simulations show that the subpopulation survival probability $p_{s}$ decreases with the degree of fragmentation for low antibiotic concentrations (figure 5*a*). This is because even if the average population density is above the survival threshold $\rho^{*}$, some subpopulations have initial population density below the threshold and are killed by the antibiotic. These subpopulations will not contribute to population growth (figure 5*b*).

### 2.6. Habitat fragmentation can decrease lag times for collective resource foraging through enzymatic degradation

Our study has focused on the case of collective enzymatic detoxification of the environment. However, microbes can modify their environment enzymatically in diverse ways, not limited to detoxification. As a contrasting example, we also modelled a microbial population, which uses enzymes to release collective nutritional benefits from its environment. Examples of such behaviour include the breakdown of sucrose by the enzyme invertase produced by yeasts [23], particulate organic matter breakdown by marine bacteria [50] and the breakdown of plant cellulose by microbial cellulase enzymes [21]. We reasoned that collective resource foraging interactions might also be affected by habitat fragmentation.

To probe this idea, we modified the model of equations (2.1) and (2.2) to represent a microbial population, which produces enzymes that release nutrients from the environment, facilitating growth. We suppose that the population cannot grow until the nutrient concentration exceeds a threshold, but once the nutrient threshold is reached it grows exponentially (electronic supplementary material, Supplementary results and figure S5). For an unfragmented population, this model shows an initial lag time while the nutrient concentration is below the threshold, followed by exponential growth once the nutrient reaches the threshold.

Repeating our simulations with systematically increasing degrees of habitat fragmentation, we observed that fragmentation decreases the lag time before population growth starts (electronic supplementary material, figure S5). This happens because subpopulations that stochastically have a higher initial population density release environmental nutrients faster, allowing them to initiate growth earlier. In this case, the ecological outcome—lag followed by growth—is the same whether or not the habitat is fragmented. Nevertheless, habitat-fragmentation-induced lag time decrease could be relevant, for example, in the case of a temporally fluctuating environment.

## 3. Discussion

Fragmented habitats are common in the microbial world. Laboratory studies on microbial ecology generally use well-mixed, unfragmented populations and the results of such studies are often extrapolated to predict microbial ecology in fragmented habitats. However, it is becoming clear that habitat fragmentation can directly influence microbial interactions, population dynamics and community ecology [12–19]; hence, there is a growing need for models that explicitly account for habitat fragmentation. Here, we develop such a model and use it to show that enzymatically mediated collective defence against a chemical toxin can be far more effective in a fragmented habitat than in a well-mixed environment.

We have used as our example the well-studied case of β-lactamase enyzme production by antibiotic-resistant bacteria. β-lactamase enzymes are of global clinical importance [20]; indeed, the World Health Organization (WHO) and US Center for Disease Control and Prevention (CDC) have identified carbapenemase-producing strains of *Pseudomonas aeruginosa*, *Acinetobacter baumannii* and Enterobacterales as critical threats [51,52]. Our study aims for fundamental insight rather than clinical predictions—for example, an infection that is known to be β-lactamase producing would generally not be treated with β-lactam antibiotics. However, our results do suggest a mechanism by which an infection that has weak β-lactamase activity, and therefore is not classified as resistant, could nevertheless survive β-lactamase treatment even at high doses in a fragmented environment such as some human tissues.

In our model, the habitat-fragmentation rescue phenomenon originates from the pockets of high local microbial density that can arise in a fragmented environment, even when the average density is much lower. Within such high-density pockets, enzyme-producing microbes are protected by their ability to rapidly degrade the antibiotic. Local pockets of high microbial density are indeed observed in soil [53], in aquatic systems [1] and in biofilm infections [54], suggesting that habitat-fragmentation rescue might be a widespread phenomenon.

In our simulations, the distribution of microbial cells among sub-volumes was the main source of stochasticity leading to habitat-fragmentation rescue. We also showed that demographic noise in birth/death dynamics can provide an alternative and (for our parameter set) similarly effective source of stochasticity. Our results support the emerging idea that habitat fragmentation increases the variability of ecological outcomes in microbial communities [12] and also strengthens the case that stochasticity can play a key role in determining the efficacy of antibiotic treatment [55–58]. Another important source of stochasticity in microbial communities is phenotypic and genotypic heterogeneity among individual microbial cells [47]. Such heterogeneity has been implicated in the dynamics of intracellular bacterial infections [59,60]; it would be interesting in future to extend our work to include heterogeneity among bacterial cells.

Collective benefits among microbes are often viewed in the context of social evolution theory, where key questions concern the establishment and maintenance of cooperators (here enzyme producers) in the presence of non-producing cheaters who do not pay the fitness cost of production [61]. In social evolution theory, fragmentation of a population increases the chance for genetically related organisms to share the benefits of cooperation (kin selection) but can also increase competition among related organisms [62]. Fragmenting a population into groups of variable size can maintain cooperators via Simpson’s paradox [63]. Our study shows that, even for a clonal population of cooperators, habitat fragmentation can strongly change the value of a collective benefit, especially in the case of toxin removal. Future work should explore the implications of this insight for mixed populations of cooperators and cheaters.

Our study is based on a simple theoretical model, although more realistic models are expected to show similar behaviour [34]. Microfluidic technology increasingly allows controlled study of the dynamics of compartmentalized small microbial populations [11–14,40,41,64], including their response to antibiotics [65]. Our study provides a baseline prediction; the reality may be more complex due to phenomena such as morphological or gene regulatory changes in response to antibiotic [66] and/or de novo evolution of further resistance.

Microbial ecology and evolution often play out in fragmented habitats, yet our strategies for understanding and controlling microbial populations are often based on extrapolation from well-mixed, unfragmented populations. Models that explicitly account for habitat fragmentation may help reveal new ecological mechanisms that are at play in naturally fragmented microbial populations.

## Data Availability

The simulation code used to generate the data presented in this article is available from Zenodo [67]. Supplementary material is available online [68].

## Footnotes

^1^
In our model, we assume that all β-lactamase enzyme is secreted. Therefore a single, isolated bacterium in an infinitely diluted environment does not gain any benefit from secreting *β*-lactamase, and the single-cell MIC in our model is equivalent to the MIC of a non-resistant strain. This is consistent with other models [29], but would not be the case in a model where a fraction of the β-lactamase is retained intra-cellularly.

^2^
For Poisson distributed initial numbers, the subpopulation number distribution has mean and variance both equal to ρV/m, so that the coefficient of variation CV in the cell number, given by standard deviation divided by mean, is $\sqrt{m/(\rho V)}$ . The CV of the cell density is equal to the CV of the cell number, since density is the number of cells divided by the constant sub-volume size.
